# Does the Video Assistant Referee (VAR) mitigate referee bias on professional football?

**DOI:** 10.1371/journal.pone.0294507

**Published:** 2023-11-27

**Authors:** Thadeu Gasparetto, Kirill Loktionov

**Affiliations:** 1 Leeds Beckett University, Carnegie School of Sport, Leeds, United Kingdom; 2 National Research University Higher School of Economics, Saint Petersburg, Russia; Reichman University, ISRAEL

## Abstract

The purpose of the paper is to check whether the introduction of the VAR system mitigated the referee bias against away teams. The dataset comprises 2279 matches played in the first tier of the Brazilian League from 2016 to 2021. We analyze 6 seasons of the first tier of the Brazilian domestic football league– 3 seasons before and 3 seasons after the introduction of the VAR technology. Potential bias is viewed through the lens of yellow cards, red cards and number of penalties awarded for both home and away clubs. A paired t-test is used to reveal potential statistical differences between pre-VAR and post-VAR periods, followed by Ordinary Least Squares regressions to inspect whether certain referee’ categories have changed their behavior after the implementation of this technology. Our empirical findings offer evidence that the referee bias is diminished, but still present.

## 1. Introduction

The sports field is actively popularized all over the world. Various competitions, from the Olympic Games to e-sports tournaments, are attracting more and more attention from a wide variety of groups of people: fans, sponsors, sports experts, scientists. The audience is interested in the persistent struggle of motivated athletes, based on the principles of fair sport and rivalry, when everything is determined by diligence and the will to win, and when any underdog can end up being the winner.

Referees have one of the key roles in any sport. Being a direct participant of the competition, the referee, in addition to his protocol duties (signaling the beginning and end of the event, fixing the result, etc.), must also ensure compliance with the sports rules. Since the athletes are under strong emotional stress and cannot give an objective assessment of game episodes independently, the referee is obliged to keep a cool head in any situation and be able to make unbiased decisions.

This study focuses on football. The most popular sport in the world is a platform for confrontation between different groups of people pursuing their own goals and interested in a certain result of the match. Since it is much more difficult to influence the whole team than one person, these people often define the referee as the object of their pressure and try to force him to make decisions in favor of one of the parties. Even though special attention is paid to the emotional preparation and stress resistance of referees, not all of them can cope successfully with such pressure.

One of the most resonant cases, when several referees were accused of bias and favoritism at once, occurred during the 2002 World Cup. The South Korean team, which co-hosted the tournament with Japan, reached the semi-finals of the tournament, where they lost to the German team. According to most experts, this was largely due to refereeing mistakes made in succession in matches against Portugal (group stage), Italy and Spain (round of 16 and quarter-final respectively) refereeing mistakes helped South Korea to go so far, which were consistently committed in favor of the hosts of the tournament in the group stage match against Portugal, in the 1/8 finals against Italy and in the quarterfinals against Spain. After the end of the tournament, Joseph Blatter, the president of FIFA at the time, publicly criticized refereeing during the championship, pointing out several serious mistakes that had a key impact on the results of the matches. The organizers of the competition also agreed with him.

The refereeing of Tom Henning Øvrebø in the 2009 Champions League semi-final between Chelsea and Barcelona stands out from other controversial refereeing in case of a single match. During the game, he had reason to appoint a penalty three times against Barcelona, but he never pointed to the 11-meter mark. As a result of the match, Barcelona reached the final of the tournament, and then won it. Øvrebø’s decisions are still being criticized by fans on social media, and the referee himself has admitted to receiving threats for several years.

In January 2022, one of the most respected German referees, Felix Zweier, temporarily suspended his career due to serious moral fatigue and the need for psychological recovery. He attributed this to severe stress and the need to recover psychologically. After the match between Bayern Munich and Borussia Dortmund, the referee was severely criticized by the players of Borussia, who lost 3:2. Pointing to several controversial decisions, the players questioned Zweier’s professional suitability, recalling that he had been involved in a major match-fixing scandal a few years earlier.

Regular episodes of psychological and even physical violence against referees, as well as recurring scandals caused by referee errors, have necessitated the use of new technologies in football. Until 2018, the referees, according to the rules, could not cancel the decision, whether it was a penalty kick, a missed goal, or a red card. At the same time, fans at the stadium could see replays of controversial episodes on large broadcast screens and quickly detect the referee’s mistake. In 2018, the International Football Association Board approved the use of the Video Assistant Referee (VAR) system in football matches. Its essence was that in addition to the main referee and his assistants, another team of referees also monitors the progress of the match via video broadcast. In case of controversial episodes, the VAR referee has the right to invite the main referee to the monitor and show him a video replay of the controversial situation. The chief arbiter, after consulting with a colleague, makes his final decision on the episode.

From the VAR system they expected, if not the complete eradication of referee errors as a phenomenon, then at least their reduction to a minimum level and a reduction in the number of scandals. However, 4 years of the system’s operation have shown that the referees continue to make controversial and even frankly erroneous decisions, despite the possibility of a detailed study of any situation in the match using video. A big controversy erupted in March 2022 around the match Everton—Manchester City in the English Premier League, where the guests won 1–0. During the game, the ball, while in the “City” penalty area, hit the defender’s hand. According to the rules, a penalty should have been awarded. However, referee Paul Tierney, after reviewing the episode and consulting with a VAR colleague, decided not to award a penalty kick. After the match, the English Football Association recognized this decision as erroneous and made an official apology to Everton. On May 22, Manchester City became the champion of England, overtaking Liverpool by 1 point. If it had been an obvious refereeing mistake, the fate of the championship could have turned out differently.

The question of whether controversial refereeing decisions are the result of random errors, or the result of systematic referee bias has formed the basis of scientific research several times not only in football, but also in other sports. Bias was analyzed through the nationality of athletes and referees, the occupancy of stadiums, and the status of rivals. Additionally, there is an ongoing debate about the use of VAR and its impacts on fairness, which several scholars have been addressing recently [1–3]. Both historical perspectives and managers perceptions are considered, but to the best of our knowledge, no empirical research has shown whether the introduction of VAR has mitigated (or not) any referee bias. Therefore, this is the gap we want to address in this research.

The purpose of this study is to find out whether there was a bias in favor of home teams before the introduction of the VAR system, and how the situation changed after its introduction in the matches of the Brazilian football league. The main hypothesis of the study was formulated as follows: in the matches of the Brazilian football league during the period under review, there is a refereeing bias in favor of home teams in terms of cards shown and penalties awarded. To achieve this, we compared two periods: pre-VAR (2016–2018) and post-VAR (2019–2021). By doing so, we aimed to evaluate the effectiveness of VAR’s introduction in promoting referee objectivity and enhancing fairness in the game. Furthermore, we can discern potential behavioral changes based on the referee category. This research contributes to a comprehensive understanding of the impact of VAR adoption on match officiation in Brazilian football. The relevance of the work is due to an attempt to compare two football periods, and to evaluate the effectiveness of the introduction of the VAR system in terms of its impact on referee objectivity.

## 2. Literature review: *Referee bias*, *its causes*, *and forms of manifestation*

Most studies of refereeing bias in football focus on yellow and red cards, the number of penalties awarded, or stoppage time. The reasons for bias and favoritism can be different: from the referee’s personal sympathy for one of the teams to racial dislike for a particular player, but the forms of its manifestation remain unchanged.

Regarding the number of cards, the main way to identify bias in this matter is to evaluate the ratio of the number of violations committed by the team to the number of warnings and removals received for it. A study on English Premier League matches between 1995 and 2002, found favoritism in favor of the home team [4]. Similar results are found in the following work inspecting matches of the Champions League and UEFA Cup [5].

Examination about red cards found that the team that remained with more players was more likely to win the match [6]. Emotional uplift and a larger number of players on the field naturally create an advantage in the game, which can often be converted into goals. This explains the active attempts of fans, players and even coaches to persuade the referee to remove the opponent after gross violations. This trend continues both in regular season matches and at the playoff stages, where the passage to the next round is decided in 1–2 matches. This conclusion is supported by another paper on the effect of suspensions on match results in the 2002 World Cup [7].

Naturally, the players do not want to earn red cards and let their team down. In order not to receive such cards, they are even ready to change their style of play, adapting to the new rules. A paper examines how card counts were affected by the introduction of a new rule prior to the 1998–1999 season requiring a direct red card for a tackle from behind without playing the ball [8]. The author found that in subsequent seasons, the number of red cards did not increase, but even decreased slightly. At the same time, there was a sharp jump in the number of yellow cards. The players, knowing about the increased probability of earning a red card, began to actively insure each other and use the tactics of “petty foul”–more often commit minor violations and receive yellow cards.

Penalties are another form to potentially identify referee bias. A study on the German Bundesliga matches in the 2000–2001 season found that home teams were awarded penalties 81% of the time they really should have been [9]. The away teams, in turn, received only 51% of penalty kicks. Thus, in half of the cases where the referees had every reason to point to the penalty mark, they did not. Another work on the Bundesliga over 12 seasons that referees tend to prefer the home side in awarding penalty kicks [10]. He also found that the most controversial and even erroneous penalties were awarded in situations where the home team was down by one goal. This can be regarded as an argument in favor of the fact that the referees thus deliberately tried to help the home teams avoid defeat.

In addition to the German championship, evidence of referee bias in favor of home teams on penalties has also been found in the English Premier League [11]. After analyzing 5244 matches, inconsistent and favorable refereeing by 50 referees was revealed. However, later research on more recent data did not confirm these results [12].

One of the main reasons for refereeing bias in relation to penalties is pressure from the stands. After examining the relationship between penalty awards and the distance from the stands to the field, authors found that referees were less likely to award mixed penalties in stadiums where fans were separated from the field by a running track [13]. Similar results were obtained in the Italian *Serie A* as well [14].

Experimental research examined whether the full stands and fan’s reaction to the events have an impact even on independent referees [15]. Authors invited several referees to watch video replays of matches in which they did not work and evaluate several episodes where it is possible to present a card or award a penalty. The first group of referees watched the video without sound, the second could hear the reaction of the stands. According to the results of the experiment, the second group of referees often make similar decisions to the referee on the field, and with more favorable actions towards the home team.

The introduction of VAR technology in professional football has been a significant development in recent years, impacting the way referees make decisions during matches. Current research on VAR in football aims to explore its effectiveness, impact on the game, and potential for improving decision-making and fairness in football. There is, indeed, evidence that this technology is very accurate [2], which would provide fairness to the game. However, the author emphasizes that its use is still controversial, corroborating another research, since English Premier League coaches currently display mixed opinions about its use [3].

Despite the significant body of research that has documented the existence of referee bias in various settings and the ongoing debate about the potential fairness VAR could bring to football matches, none of these studies has empirically investigated whether the introduction of VAR has actually mitigated referee bias. This gap in the literature highlights the need for further research to explore the impact of VAR on the fairness and accuracy of refereeing decisions. To address this gap, the present study aims to examine the effectiveness of VAR in reducing referee bias in football matches. By doing so, this study seeks to contribute to the ongoing discussion about the role of technology in improving the objectivity and consistency of refereeing decisions in sport.

## 3. Methods

### Setting

The introduction of the Video Assistant Referee (VAR) system in Brazilian football took place in a quarter-final match of the Brazilian Cup in 2018. However, the VAR’s integration into the top-tier league, *Campeonato Brasileiro Série A*, began in the 2019 season. This technological innovation aimed to enhance the accuracy of officiating by allowing referees to review critical decisions such as goals, penalties, and red cards.

For the present study, we compiled a panel dataset comprising data from 2,279 matches in the Brazilian Serie A (first tier) across the seasons from 2016 to 2021. Our aim was to establish a balanced panel, dividing the dataset into two equal periods of three seasons each. The primary objective is to investigate potential variations in the allocation of yellow and red cards, as well as penalty kicks, by referees for both home and away teams between these distinct timeframes. Given the objective of enhancing fairness in the game through the introduction of VAR technology, we empirically assess its impact within the context of Brazilian football. The dataset is available as a Supporting Information file.

### Empirical strategy

The empirical strategy initiates with a paired t-test, a statistical method comparing the averages of yellow cards, red cards, and penalty kicks before and after the introduction of VAR. This test assesses whether there are statistically significant differences in these indicators between the two periods. A significant result implies that there was a notable change in referee decisions, with the direction of change determined by the sign of the t-statistic. For instance, a negative t-statistic indicates a decrease in card or penalty kick assignments, while a positive t-statistic suggests an increase.

Later, panel data Ordinary Least Squares (OLS) regressions are carried out. These econometric models aim to reveal whether specific referee categories altered their behavior following the introduction of VAR. By examining the relationships between referee categories (*R*), control variables (*CV*), and the outcomes of interest (yellow cards, red cards, and penalty kicks), these models provide insights into the impact of VAR on referee decisions. The coefficients (*θ*, *δ*, *ϑ*) associated with referee categories in these models will indicate whether and to what extent certain referee groups adjusted their officiating tendencies in the post-VAR period. The general models are presented below:

$$y_{it}=\beta_{0}+\theta R_{it}+\beta_{1}CV_{it}+\varepsilon_{it}$$

$$r_{it}=\gamma_{o}+\delta R_{it}+\gamma_{1}CV_{it}+\varepsilon_{it}$$

$$p_{it}=\alpha_{o}+\vartheta R_{it}+\alpha_{1}CV_{it}+\varepsilon_{it}$$

Where:

*i* = a given match

*t* = a given season

*R* = matrix of referee categories

*CV* = control variables

*ε*_*it*_ = error term

Three dependent variables are regressed: yellow cards (*y*), red cards (*r*), and penalty kicks (*p*). All models were regressed using identical sets of explanatory and control variables, which allows comparisons between them. The variables of interest are in the matrix of main referee categories (*R*). Main referees are sorted into three categories: CBF, MTR and FIFA, from the least to the most experienced ones. The first group is used as base/reference category in the equations. Our goal is to check whether referees from any of these categories have changed their behavior after the introduction of VAR.

We incorporate several control variables (*CV*) to enhance the precision and reliability of our analysis. These control variables, including referee age, square difference in the win probabilities of home and away team, league table positions for both home and away teams, and average statistics such as yellow cards, red cards, and penalty kicks assigned to the home team in previous matches, serve multiple crucial purposes. Firstly, they aid in the mitigation of potential confounding factors that may influence the allocation of decisions by referees. Secondly, these variables allow us to isolate and estimate the specific impact of VAR introduction on referee bias while accounting for extraneous influences. Additionally, fixed effects for both home and away clubs, as well as referee fixed effects, are integrated into the modeling to control for unobservable characteristics unique to the home and away clubs and individual referees. These fixed effects are integrated into the modeling to also mitigate potential confounding effects arising from the influence of these factors on the observed outcomes, ensuring the reliability of the model’s estimates.

The division into separate pre-VAR and post-VAR periods is essential to isolate and examine the distinct effects of VAR introduction on key indicators. This approach facilitates a comprehensive understanding of the impact of VAR adoption on the observed changes in the studied outcomes

## 4. Results

Table 1 below shows the average number of yellow cards, red cards, and penalties–assigned to home and away team. T-value and significance are also displayed in the table.

**Table 1:** Average cards and penalties pre- and post-VAR introduction.

	Yellow Card		Red Card		Penalty Kicks	
	Home Team	Away Team	Home Team	Away Team	Home Team	Away Team
**Mean (Pre-VAR)**	2.16	2.55	0.14	0.22	0.12	0.08
**Average (VAR)**	2.24	2.30	0.14	0.25	0.15	0.10
**t-value**	-1.42	4.02	-0.29	-1.49	-1.91	-1.65
**Pr (|T| > |t|)**	0.1549	0.0001***	0.7726	0.1351	0.0567*	0.0993*

* *p* < 0.10,

** *p* < 0.05,

*** *p* < 0.01.

Table 1 presents a summary of the average yellow cards, red cards, and penalty kicks for both home and away teams in the pre-VAR (2016–18) and post-VAR (2019–21) periods. The mean number of yellow cards for home teams increased slightly from 2.16 in the pre-VAR period to 2.24 in the post-VAR period, although this difference was not statistically significant (t = -1.42, p = 0.1549). However, the mean number of yellow cards for away teams decreased from 2.55 in the pre-VAR period to 2.30 in the post-VAR period, with a statistically significant difference (t = 4.02, p < 0.01). The observed decrease in the mean number of yellow cards for away teams in the post-VAR period, along with the statistically significant difference, suggests that VAR might have played a role in mitigating referee bias against away teams, providing evidence of a reduced number of yellow cards shown to them.

Additionally, there was only a marginal change for both home and away teams regarding red cards. The mean number of red cards for home teams have not changed between period, and for away teams, it increased from 0.22 to 0.25. These differences were not statistically significant, with t-values of -0.29 (p = 0.7726) for home teams and -1.49 (p = 0.1351) for away teams.

In terms of penalty kicks, home teams saw an increase in the mean number of penalties awarded from 0.12 in the pre-VAR period to 0.15 in the post-VAR period, although this difference was not statistically significant (t = -1.91, p = 0.0567). Similarly, away teams experienced a slight increase from 0.08 to 0.10 in the mean number of penalty kicks, with weak evidence of a potential difference (t = -1.65, p = 0.0993). The observed increase in the mean number of penalties for both home and away teams, although not statistically significant (only at 10%), may indicate a tendency toward a higher number of penalties being assigned in the post-VAR period. Referees now have the support of VAR to identify penalty kicks and reconsider their initial decisions, which could contribute to this observed trend.

Overall, the introduction of VAR appeared to have a more pronounced impact on the number of yellow cards, leading to a statistically significant decrease for away teams. However, the effects on red cards and penalty kicks were less evident, with no significant changes observed for home teams and only weak indications of potential differences for away teams. It is essential to consider these findings within the broader context of VAR’s influence on disciplinary actions in football matches.

### 4.1. Regression analysis: Exploring referee category influence

In order to further examine the potential factors influencing the observed changes in disciplinary actions following the introduction of VAR, we conducted Ordinary Least Squares (OLS) regression analyses. Our aim was to explore whether specific referee categories played a significant role in driving these changes. By examining the impact of referee characteristics, we can gain insights into whether certain groups of referees were more inclined to adapt their officiating style in response to VAR implementation.

We performed a total of 12 regression models. There are three dependent variables (i.e., yellow cards, red cards, and penalty kicks), which were regressed twice–pre-VAR and post-VAR introduction–for both home and away clubs. Table 2 shows the six models for home clubs, while Table 3 the models for away clubs.

**Table 2:** Regression outputs for yellow cards, red cards, and penalties for the home teams.

	Pre-VAR (2016–2018)			Post-VAR (2019–2021)		
Estimators	Yellow cards	Red cards	Penalties	Yellow cards	Red cards	Penalties
MRC = FIFA	-0.81 * (0.41)	3,1e-02 (7.5e-02)	0.17 (0.14)	-8.7e-0.1 * (4.3e-01)	-0.1 (0.11)	-0.01 (0.13)
MRC = MTR	-1.35 (0.77)	-1e-01 (1.7e-01)	0.04 (0.21)	-1.6e * (8.5e-01)	-0.7 * (0.35)	-0.44 (0.37)
CV	Yes	Yes	Yes	Yes	Yes	Yes
*Home Team FE*	Yes	Yes	Yes	Yes	Yes	Yes
*Away Team FE*	Yes	Yes	Yes	Yes	Yes	Yes
*Main Referee FE*	Yes	Yes	Yes	Yes	Yes	Yes
*R2*	0.1901	0.1168	0.1175	0.1539	0.1434	0.1224
*Adjusted R2*	0.0928	0.0107	0.0114	0.0607	0.049	0.0257
*Observations*	1139	1139	1139	1140	1140	1140

Robust Standard errors in parentheses.

* *p* < 0.10,

** *p* < 0.05,

*** *p* < 0.01.

**Table 3:** Regression outputs for yellow cards, red cards, and penalties for the away teams.

	Pre-VAR (2016–2018)			Post—VAR (2019–2021)		
Estimators	Yellow cards	Red cards	Penalties	Yellow cards	Red cards	Penalties
MRC = FIFA	-1.12 * (0.48)	1.5e-2 (1.2e-01)	-0.11 (0.09)	0.003 (0.52)	-0.04 (0.14)	0.02 (0.11)
MRC = MTR	-1.58 (0.87)	-1e-01 (2.3e-01)	0.21 (0.18)	2.2 (1.45)	-0.01 (0.37)	0.007 (0.32)
CV	Yes	Yes	Yes	Yes	Yes	Yes
*Home Team FE*	Yes	Yes	Yes	Yes	Yes	Yes
*Away Team FE*	Yes	Yes	Yes	Yes	Yes	Yes
*Main Referee FE*	Yes	Yes	Yes	Yes	Yes	Yes
*R2*	0.1424	0.1134	0.1454	0.1771	0.1138	0.1051
*Adjusted R2*	0.0394	0.0069	0.0427	0.0864	0.0161	0.0065
*Observations*	1139	1139	1139	1140	1140	1140

Robust Standard errors in parentheses.

* *p* < 0.10,

** *p* < 0.05,

*** *p* < 0.01.

The estimators MRC = FIFA and MRC = MTR represent different referee categories and provide insights into their influence on disciplinary actions. In the pre-VAR period, FIFA referees were assigning significantly fewer yellow cards to home teams than their counterparts (MTR and CBF). No differences in red cards and penalty kicks are observed. However, after the introduction of VAR, both FIFA and MTR referees assigned statistically fewer yellow cards to home teams than the base category–the less experienced referees. Additionally, MTR referees have also shown a significantly reduced number of red cards.

Regarding the cards and penalty kicks assigned to away teams, one can notice that the only statistically significant variable is the yellow cards in the pre-VAR period, which indicates that the most experience referees have been showing a fewer number of yellow cards to away teams than their counterparts. Nonetheless, after the introduction of the VAR, such a difference is no longer observed, implying that all categories are currently refereeing at similar manner.

### 4.2. Discussion

Overall, the average numbers indicate some differences in all three variables analyzed. Brazilian referees tend to show fewer yellow and red cards to home teams than to away teams, as well as award a higher number of penalties to home than away teams. It could be interpreted as some sort of referee bias–indeed, it has been previously detected in Brazil, where referees consistently exhibited a bias in favor of home teams by allocating additional time in closely contested matches when the home team was losing [15]. Nonetheless, the home advantage, often observed on professional football [16–18], can be driven these figures, as well as such numbers might also be affected by the crowd support [19]–which would be a bias by itself. Previous research had already evidenced the presence of referee bias towards home clubs [20,21] and its potential influence on home advantage [11]. Once the researched period may reflect a *natural experiment*, we ran paired t-test to reveal potential statistical differences between pre- and post-VAR as well as regression models to check whether certain referee categories would be influencing the potential changes.

Our empirical results indicate that after the introduction of VAR, away clubs have been awarded a fewer number of yellow cards, which might be interpreted as a sign of a mitigation of referee bias. Since the referee’s performances are now under larger scrutiny, this external pressure could be driven to less biased behavior against away teams, which lead into a reduced number of yellow cards to those teams. On the other hand, the introduction of this new refereeing technology did not imply a significant change in the number of red cards assigned, although there is some evidence that the number of penalty kicks have slightly increased for both home and away clubs. We assume that the opportunity to revise such controversial moments may have contributed to this higher number of penalties observed.

Former study discusses VAR as an accurate tool for decision-making on football [2], but there is evidence that football managers’ perceptions on this regard are still mixed [3]. We do acknowledge that the current method employed does not allow us a proper identification of the reasons for the changes observed. However, once the introduction of the VAR technology is the only noted alteration between both periods, it is conceivable to assume that these changes could have happened due to the opportunity to revise controversial moments (i.e., red cards and penalty kicks), as well as due to the external pressure referees are experiencing regarding their decisions. In this sense, our empirical findings suggest that the most experienced referees (i.e., FIFA and MTR) could have adjusted their refereeing behavior, which lead into a slightly mitigation of referee bias against away teams–particularly regarding the significant reduction of yellow cards awarded to them. Therefore, we believe that our empirical findings can contribute to this discussion about impacts of the introduction of VAR, although encouraging further research in different settings.

It should be noted that most of the VAR period discussed in the article coincided with the time of the Covid-19 pandemic, which potentially changed the way sports competitions were held around the world. For almost one and a half seasons, the matches were held in empty stadiums, and therefore the pressure on the referees was greatly reduced from the fans. Some recent papers show that empty stadiums have reduced referee bias on football [22–26], while a recent paper claim that VAR significantly reduced the home advantage in the German Bundesliga but did not impact the referee bias in that setting [27]. Like our research, none of those papers have disentangled the VAR and Pandemic effects. We do recognize this as a limitation of our research, but we believe it does not undermine the analysis performed. It is possible that one factor may be influencing another (such as VAR affecting empty stadiums or vice versa). Nonetheless, our primary objective, which is to evaluate the reduction of referee bias, remains valid. Therefore, we encourage further research to compare our empirical outcomes with prior studies on empty stadiums but inspecting larger datasets and employing diverse econometric approaches–this would allow for a precise identification of effects from empty stands and VAR separately. Consequently, one would be able to better assess the individual contributions of various factors in reducing referee bias against away football teams.

Lastly, it is important to acknowledge that our analysis of potential referee bias has primarily focused on yellow and red cards, as well as penalty kicks. While these are significant indicators, professional sports involve numerous on-match elements that could potentially contribute to bias. However, due to the scope of a single paper and lack of available data, we have not explored these other factors comprehensively (e.g., fouls and free kicks, time management, verbal communication, VAR usage, etc.). Further research is essential to identify potential negative behaviors towards participants and, equally important, to develop strategies to mitigate unfairness within professional sports.

## 5. Conclusion

This research examined the existence of referee bias on Brazilian football and whether the introduction of VAR on professional football has reduced such bias through a reduction on the number of cards and penalties earned by home and away teams. In a nutshell, the empirical results indicate favorable decision-making from referees towards home teams but show evidence that it has been slightly mitigated after the introduction of the VAR technology, particularly reducing the number of yellow cards awarded to away teams.

We acknowledge that our econometric model cannot identify the reasons for the reduction of the bias. The support provided by the video referee is likely to impact such decline, but other elements might be shaping it. The psychological pressure that referees are facing now–due to a large coverage of their actions–could be a potential explanation for a more careful refereeing style. Further research inspecting a longer dataset is encouraged. Moreover, the analysis of similar features in different scenarios (e.g., other professional football leagues worldwide, different sport disciplines, etc.) shall be able to offer a more conclusive picture of the potential of VAR in mitigating referee bias.

In conclusion, reducing referee bias in football is a complex task that requires a multifaceted approach. Our findings suggest that increasing transparency in all aspects related to the work of referees could potentially reduce errors and scandals. For instance, introducing post-match interviews with VAR referees and their assistants to explain the rationale behind their decisions publicly could increase the trust of fans about referees’ decisions. Additionally, strengthening the sanctions for gross errors can be a powerful mechanism for referees to double-check their decisions and resist pressure from the stands. In addition, educating and training referees to better understand and manage their biases can also be a valuable tool in the fight against referee bias. Further research in this area can provide additional insights into the nature of referee bias and effective strategies for addressing it.

## Supporting information

S1 Data (XLSX)
